# SCARB1 downregulation in adrenal insufficiency with Allgrove syndrome

**DOI:** 10.1186/s13023-023-02763-w

**Published:** 2023-06-19

**Authors:** Giacomo Bitetto, Gianluca Lopez, Dario Ronchi, Alessandra Pittaro, Valentina Melzi, Erika Peverelli, Fulvia Milena Cribiù, Giacomo P. Comi, Giovanna Mantovani, Alessio Di Fonzo

**Affiliations:** 1 Neurology Unit, IRCCS Foundation Ca’ Granda Ospedale Maggiore Policlinico, Via Francesco Sforza 35, 20122 Milan, Italy; 2 Division of Pathology, IRCCS Foundation Ca’ Granda Ospedale Maggiore Policlinico, University of Milan, Milan, Italy; 3 Department of Clinical Sciences and Community Health, University of Milan, Milan, Italy; 4 Endocrinology Unit, Fondazione IRCCS Ca’ Granda Ospedale Maggiore Policlinico, Milan, Italy

**Keywords:** Allgrove syndrome, Adrenal cortex, Adrenal insufficiency, PKA, SCARB1

## Abstract

**Background:**

Allgrove disease is a rare genetic syndrome characterized by adrenal insufficiency, alacrimia, achalasia and complex neurological involvement. Allgrove disease is due to recessive mutations in the *AAAS* gene, which encodes for the nucleoporin Aladin, implicated in the nucleocytoplasmic transport. The adrenal insufficiency has been suggested to rely on adrenal gland-ACTH resistance. However, the link between the molecular pathology affecting the nucleoporin Aladin and the glucocorticoid deficiency is still unknown.

**Results:**

By analyzing postmortem patient’s adrenal gland, we identified a downregulation of Aladin transcript and protein. We found a downregulation of Scavenger receptor class B-1 (SCARB1), a key component of the steroidogenic pathway, and SCARB1 regulatory miRNAs (mir125a, mir455) in patient’s tissues. With the hypothesis of an impairment in the nucleocytoplasmic transport of the SCARB1 transcription enhancer cyclic AMP-dependent protein kinase (PKA), we detected a reduction of nuclear Phospho-PKA and a cytoplasmic mislocalization in patient’s samples.

**Conclusions:**

These results shed a light on the possible mechanisms linking ACTH resistance, SCARB1 impairment, and defective nucleocytoplasmic transport.

## Introduction

The adrenal gland is a complex endocrine gland composed of two developmentally unrelated tissues, an outer layer of adrenal cortex and an inner layer of adrenal medulla [1].

The adrenal cortex is an important site of synthesis for three different classes of steroid hormones. Mineralocorticoids (aldosterone) are produced by the cells of the adrenal zone glomerulosa, which is the outermost layer, glucocorticoids (cortisol and corticosterone) are synthesized in the adrenal cortical zone fasciculata, and androgens (androstenedione and dehydroepiandrosterone) in the inner zone reticularis [2, 3].

Aldosterone synthesis mainly responds to the renin-angiotensin regulatory pathway, whereas adrenal cortical zona fasciculata and reticularis produce hormones in response to adrenocorticotropic hormone (ACTH) stimulation [4–6].

ACTH exerts its role in promoting steroidogenic cell growth, leading to adrenal cellular hypertrophy and hyperplasia, and stimulates acute and chronic adrenal response [7]. The ACTH stimulation is provided by cyclic-AMP which increases in the adrenal cells and the consequent cyclic AMP-dependent protein kinase (PKA) cleavage and phosphorylation. Phosphorylated PKA translocates from cytoplasm to the nucleus where, among several different effects, it induces the transcription of the scavenger receptor class B-1 (SCARB1 or SR-B1) [7, 8]. SCARB1 is a plasma membrane receptor for high-density lipoprotein cholesterol (HDL), which mediates cholesterol transfer to and from HDL. Once transcribed, SCARB1, stimulating cellular cholesterol uptake, promotes steroid production [1, 2, 6].

Two microRNAs, miRNA-125a and miRNA-455, are known to regulate SCARB1 expression binding to specific sites in the 3′ UTR of SCARB1 mRNA [9]. In steroidogenic cells, miRNA-125a and miRNA-455 overexpression inhibit SCARB1-related steroid hormone synthesis, on the contrary, a downregulation of those miRNAs increases the amount of SCARB1 protein on the cell surface [1].

The complex pathway from ACTH stimulation to steroid hormones production can be impaired at different points. For example, mutations in the key components of steroidogenesis can lead to ACTH unresponsiveness and adrenal insufficiency [6]. Hereditary unresponsiveness to ACTH, or familial glucocorticoid deficiency (FGD), can present with symptoms of acute adrenal crisis or with chronic adrenal insufficiency, with mineralocorticoids production being typically conserved [2]. Several autosomal recessive causes of FGD have been identified, for example, mutations in the gene of the ACTH receptor, melanocortin-2 receptor (MC2R), caused FGD type 1, or in the melanocortin-2 receptor accessory protein (MRAP) are classified as FGD type 2 [10–12]. While all the FGD syndromes are clinically very similar, a more complex disease results from the mutation of a gene encoding a Nucleoporin (Nup), the Allgrove syndrome (ORPHA:869) [13].

Allgrove syndrome is caused by the mutation of the *AAAS* gene, causing adrenal insufficiency, achalasia, alacrimia, and neurological impairment [13–15]. The *AAAS* gene, on chromosome 12q13, is expressed as two differentially spliced isoforms, which differ in the inclusion of exon 6. The encoded protein is the WD-repeat-containing protein Aladin, one of the nucleoporins forming the nuclear pore complex (NPC), whose complex function is still under debate[16, 17]. Allgrove disease appears usually in childhood, with a plethora of different clinical presentations. Glucocorticoid deficiency and adrenal crisis represent the most common and life-threatening characteristics [15]. Aladin protein (like AAAS mRNA) seems ubiquitously expressed with predominance in the adrenal gland and in the central nervous system [18, 19]. Aladin is a cytoplasmatic component of the NPC, a protein complex of more than 30 Nups, fundamental for the transport of matter, energy, and information between the cellular nucleus and cytoplasm [16]. Despite the detailed role of some Nups and their connection to pathology have been clarified, the specific function of Aladin remains elusive [20]. Nucleocytoplasmic transport defects of ferritin heavy chain, DNA ligase I and aprataxin have been detected in vitro due to Aladin damages suggesting cell susceptibility to oxidative stress after Aladin impairment [21–24]. Moreover, in H295R adrenocortical tumor cells, AAAS knockdown seems to reduce protein expression of steroidogenic acute regulatory protein StAR and P450c11β, components of the steroidogenic pathway [22].

Nevertheless, the precise mechanism that leads to a tissue-specific involvement degeneration in Allgrove syndrome and the role played by Aladin protein in adrenal insufficiency need to be better defined. In particular, the consequences of Aladin impairment on specific molecules of ACTH signaling in adrenal cells, such as MC2R and SCARB1, are still unknown.

Here, starting from the autoptic tissues of a patient with Allgrove syndrome [25], carrying the homozygous mutation c.464G > A (p.Arg155His) in the *AAAS* gene, we show the related pathology at the cortical adrenal gland level and provide evidence of an adrenal strong reduction of MC2R and SCARB1. These results stimulate further studies implying ACTH-signaling pathway and nucleocytoplasmic transport impairment linked to adrenal insufficiency, suggesting new topics for future research and unexplored therapeutic perspectives.

## Materials and methods

### Genetic and cellular analyses

Informed consent was obtained from the patient. The relevant ethical authorities, “Comitato Etico Milano Area 2”, approved this study. The mutation analysis and clinical details are reported in a previous study [25]. RNA was extracted from frozen autoptic tissues. Independent RNA isolations were performed from cortical adrenal gland using the Reliaprep RNA cell miniprep system (Promega) according to the manufacturer’s instructions. RT-PCR assays were used to evaluate the inclusion of exon 6 within AAAS transcripts. For quantitation, we performed SYBR green qRT-PCR relative quantification analysis on a 7500 Real-Time PCR System (Applied Biosystems). The deltadeltaCt method was used to calculate the relative quantification (RQ) values of full-length AAAS transcript variant 1 (AAAS-v1, NM_015665.6) and deltaE6 AAAS transcript variant 2 (AAAS-v2, NM_001173466.1), after normalization to multiple housekeeping genes (ACTB encoding beta-actin and rRNA 18S).

We also performed RNA isolation of SCARB1 from affected and control tissues, using Agilent Total RNA Isolation Mini Kit according to manufacturer’s instructions. RT-PCR and SYBR green qRT-PCR relative quantification analyses were executed using the forward primer *AGAATAAGCCCATGACCCTGAA* and reverse primer *ACCGTGAAGAGCCCAGAGTCG*. RQ values of SCARB1 transcript of patient and control were calculated by deltadeltaCt method after normalization ACTB and rRNA 18S.

### MiRNA quantification

To prepare the RT master mix the TaqMam MicroRNA Reverse Transcription Kit was used.

Three replicates were performed for each reaction including in the plate a small RNA assay for each DNA sample, the endogenous control assay and NTC reaction were used to evaluate background signal.

### Western blot analysis

Protein aliquots were extracted from fibroblasts and autoptic tissues using denaturating (SDS) and reducing (Beta-mercaptoethanol) agents. Independent protein extractions were performed from cortical adrenal gland avoiding the adrenal medulla. Protein lysates of samples overexpressing wild-type and mutated Aladin were separated on a 4–12% polyacrylamide gel and blotted on a nitrocellulose membrane (Whatman). Subcellular fractions of tissues (autoptic cortical adrenal gland) were obtained using Protein Fractionation Kits (Thermofisher).

Samples were probed with the following antibodies: Anti-AAAS TA808612 (Origene) mouse (1:1000), Actin A2066 (Sigma) rabbit (1:1200), Lamp1 AB25630 (Abcam) mouse (1:800), Lamin a/c 2032S (Cell signaling) rabbit (1:800–1000), α-tubulin 3873S (Cell signaling) mouse (1:800–1:1000), GAPDH G8795(Sigma) rabbit (1:1000), PKA EP2606Y (Abcam) rabbit (1:5000) as loading and fractionating controls.

### Morphological and immunohistochemical studies

For morphological and immunohistochemical evaluations, entire adrenal glands were collected during autopsy according to standard procedures. 2.5 μm-thick sections from formalin-fixed, paraffin-embedded adrenal gland tissue samples of the patient and autoptic specimens from two age-matched controls were stained with hematoxylin–eosin, Aladin (TA808612, 1:50, Origene, mouse), Lamin A/C (E1, 1:1000, Santa Cruz, mouse), MC2R (EPR8386, 1:100, Abcam, rabbit).

### Statistical analyses

Due to the rarity of Allgrove syndrome and considering the availability of autoptic specimens of a single patient, to reduce the individual variability we used samples from two biological controls, and all experiments were performed in triplicate. All quantitative data are expressed as mean ± standard deviation of the mean of the triplicates for each sample.

Experiments involving two biological groups were analysed by two-tailed unpaired Student's *t*-test. Significance * = *p* < 0.05.

## Results

The patient’s clinical history and diagnostic exams revealing the homozygous c.464G > A (p.Arg155His) mutation in the *AAAS* gene, causing Allgrove syndrome, have been previously reported [25].

Briefly, the patient complained at the age of 47 years for dysphagia caused by achalasia, and then progressive gait impairment since the age of 59. He developed severe polyneuropathy. Schirmer’s test revealed hypolacrimation.

The patient suffered from hypoglycaemic and hypotensive episodes suggesting an impaired hypothalamus-hypophysis-adrenal axis. He recalled past episodes of loss of consciousness while transferring from lying to standing, suggesting orthostatic hypotension, which was confirmed by the clinical evaluation. The patient had also presented episodes of symptomatic hypoglycaemia [25]. ACTH plasma level was measured at the superior limit of the range at the ACTH dosage (38.8 pg/ml). Hypocortisolism was confirmed by repeatedly low cortisol levels at basal condition and after 250 μg ACTH stimulation test which required replacement therapy with cortisone acetate.

### Transcript analyses

The mutation lies on exon 6 of the *AAAS* gene transcript AAAS-v1 while it is absent in the gene transcript AAAS-v2 [25].

**Fig. 1:**
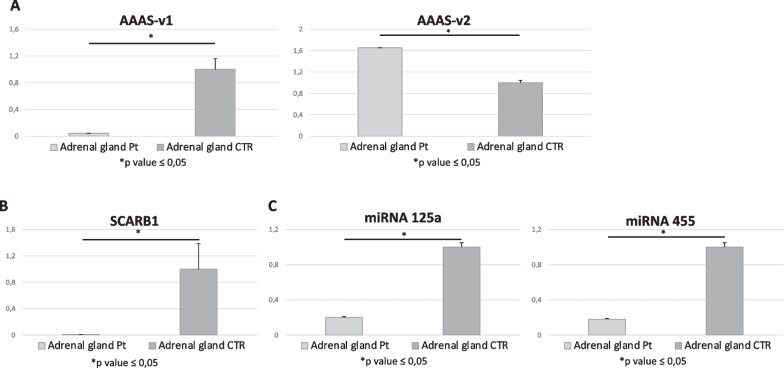
**A** The difference in AAAS-v1 and AAAS-v2 transcript levels in the adrenal gland of the patient with Allgrove diesease (Pt) and two age-matched controls (CTR). **B** SCARB1 transcript analysis by RT-PCR in cortical adrenal gland of patient (Pt) and controls (CTR). **C** MiRNA-455 e miRNA-125a concentrations in cortical adrenal gland. Both miRNAs are significantly decreased in patient

Amplification and sequencing analysis of cDNA (retrotranscribed from RNA from the patient’s tissues) confirmed the presence of the mutation at the transcript level. The two transcript isoforms were studied in tissues by RT-PCR. When comparing the adrenal gland transcript expression of the patient with controls, a significant reduction of AAAS-v1 transcript level was found. Differently, AAAS-v2 was significantly upregulated (Fig. 1-A), in line with the observation on the central nervous system [25].

In order to evaluate the impact of the impaired Aladin on adrenal gland function in the patient, SCARB1 transcript was analysed by RT-PCR in RNA from the tissues of the patient and controls.

The expression was significantly reduced in the patient’s adrenal gland compared with the controls (Fig. 1-B).

Moreover, to further investigate the epigenetic mechanism regulating SCARB1 expression, two miRNAs known to regulate SCARB1, miRNA-455 e miRNA-125a, were quantified in adrenal gland. Both miRNAs were found to be significantly decreased in the patient (Fig. 1-C).

### Morphological and immunohistochemical studies

Macroscopic examination of the patient’s adrenal gland revealed severe bilateral atrophy in comparison to the autoptic adrenal gland from an aged-matched healthy control. The patient’s adrenal glands appeared smaller and more shriveled than normal glands with a diffuse decrease in size and weight. The surface of the glands was smooth, with a loss of the normal nodularity. The color of the glands was yellowish due to the presence of fat.

**Fig. 2:**
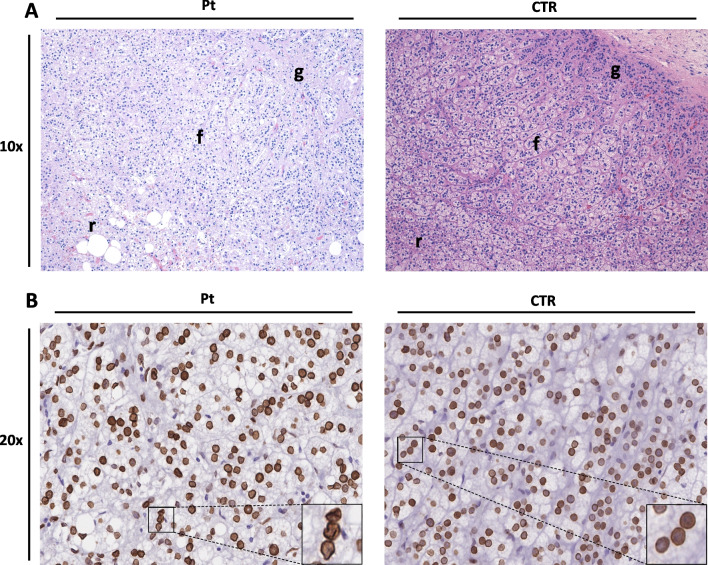
**A** Hematoxylin–Eosin staining of cortical adrenal gland from patient (Pt) and controls (CTR) (10 × magnification). The image shows differences in cytoarchitecture of cortical adrenal zones glomerulosa (g), fasciculata (f) and reticularis (r) in patient’s tissue with areas of atrophy, cellular scarcity and cellular irregularities characterized by fibro-lipidic substitution, especially of the inner layers. **B** Immunohistochemistry of adrenal gland using Lamin A/C antibody shows irregular shape of nuclear lamina in patient’s cortical zones and a normal staining in the control (20 × magnification). The boxes represent a higher magnification further highlighting the aberrant nuclear membrane shape in patient’s sample

Microscopical examination of the adrenal cortex showed the normal cytoarchitectural sub-compartmentalization in glomerular, fasciculata and reticularis zones both in patient and in control. However, patient’s tissues displayed several areas of fibro-lipidic substitution with a vacuolated appearance, especially of the inner layers. Moreover, several areas of reduced glandular cell volume with disorganized and atypical cell arrangements were detected (Fig. 2-A).

Lamin A/C staining revealed a diffuse irregularity of the nuclear membrane shape in the patient’s cortical adrenal gland (Fig. 2-B).

**Fig. 3:**
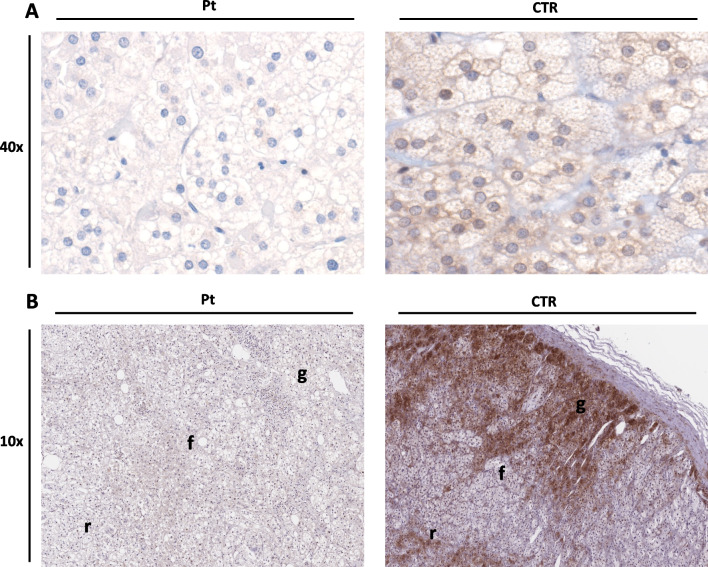
**A** Immunohistochemistry of adrenal gland using Aladin antibody shows a significant reduction of Aladin staining in all patient’s cortical zones (Pt) and a normal staining in the controls (CTR) (40 × magnification). **B** Immunohistochemistry of adrenal gland using of the membrane ACTH receptor, MC2R, antibody shows a strong cytoplasmic reduction in patient’s tissues an intense staining in several areas of the control’s cortical adrenal gland (10 × magnification)

The staining of Aladin was significantly reduced in patient adrenal tissues. An intense mostly perinuclear signal was detected in all three adrenal sub-regions of the control, while it was almost absent in the patient in all three cortical layers (Fig. 3-A).

To further explore the role of Aladin in the pathway of adrenal cortex insufficiency, we analysed the expression of MC2R. Interestingly, the immunohistochemistry showed a strong cytoplasmic reduction in the patient’s cortical adrenal gland (Fig. 3-B).

### Western blot studies

**Fig. 4:**
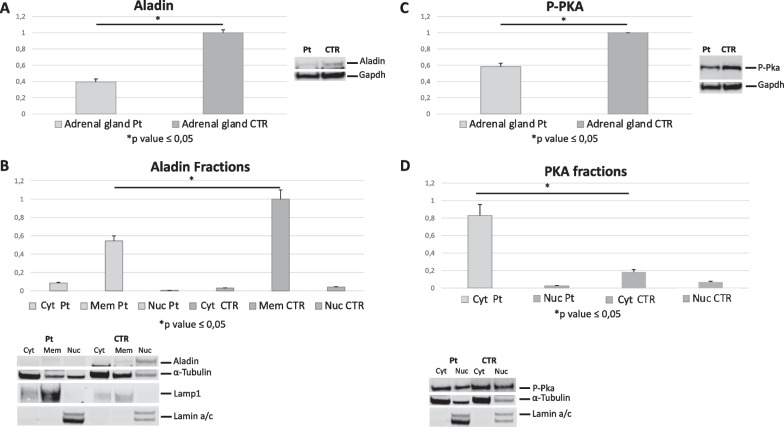
**A** Reduction of Aladin protein amount in total lysate of patient’s cortical adrenal gland (Pt). **B** Reduction of Aladin protein in membrane fraction from patient’s cortical adrenal gland with a slight increasing in patient’s cytoplasmic fraction. **C** Reduction in P-PKA concentration in cortical adrenal gland from patient compared to two controls (CTR). **D** Increase of P-PKA protein in cytosolic fraction from patient cortical adrenal gland compared to controls and not significative reduction in P-PKA protein amount in patient’s nuclear fraction

Aladin protein was significantly reduced in protein lysates extracted from patient’s cortical adrenal gland (Fig. 4-A).

Aladin, which normally localizes mainly in the membrane fraction, was reduced in patient’s membranes and it is slightly increased in the cytosol (Fig. 4-B).

P-PKA was found to be significantly reduced in patient’s cortical adrenal gland total lysate (Fig. 4-C).

P-PKA protein was found slightly reduced in patient’s nuclear fraction compared to controls and significantly increased in patient’s cytosolic fraction (Fig. 4-D).

## Discussion

Allgrove syndrome displays glucocorticoid deficiency as a cardinal feature, however, the link between the mutated protein Aladin and the adrenal gland dysfunction is largely unexplored.

Some reports suggested an important role of oxidative stress in adrenal gland cell lines knockdown for Aladin [23, 24].

In order to clarify the role of Aladin in the steroidogenic pathway, we examined the adrenal gland of a patient with Allgrove syndrome, a rare disease due to Aladin loss of function.

We first observed a macroscopic adrenal gland volume reduction respect to the controls, with a specific atrophy of zone fasciculata and reticularis accompanied by a strong reduction of Aladin staining and significant irregularities of nuclear membrane in the patient. Moreover, we confirmed the significant lower amount of Aladin protein in Western Blot of total lysates and subcellular fractions from the patient’s cortical adrenal gland. In line with these observations, we previously demonstrated that Aladin was reduced in amount, and slightly increased in the cytoplasm, also in neurons and fibroblasts of the same patient [25].

The c.464G > A mutation is predicted to lead to a missense p.Arg155His change at the protein level.

Here, we analyzed the expression of AAAS transcripts from adrenal gland. Patient’s tissues displayed a reduction of AAAS-v1 transcript expression. This finding opens two possible scenarios: either the c.464G > A substitution induces an aberrant transcription with intron retention, that eventually undergoes degradation through Nonsense Mediated Decay (NMD), or it may increase the affinity of the mutated transcript (AAAS-v1) for RNA interference molecules. As we previously demonstrated in the same patient’s fibroblasts [25], the lack of transcript changes, after NMD-inhibition with cycloheximide, makes the first hypothesis less probable. Further experiments will explore the role of small interference RNAs as possible causes of transcript 1 downregulation. Interestingly, patient’s tissues displayed marked AAAS-v2 over-expression. This result could be explained by a compensatory mechanism in these impaired cells.

Combined with the observation of oxidative stress, the pathological examination suggested a higher susceptibility of these specific regions to cell death. The degenerative process involving specifically this region may be related to the adrenal insufficiency of this syndrome. This hypothesis would explain the observed atrophy of the adrenal gland regardless of the ACTH plasma levels.

However, other possible functional reasons related to the role of the Nup Aladin in the nucleocytoplasmic transport and its impact on the ACTH-signaling cascade should be taken into account. To further support this line of reasoning, a downregulation of proteins implicated in steroidogenesis, such as steroidogenic acute regulatory protein StAR and P450c11β, has been reported in vitro [22].

In this view, we tested the possibility that the ACTH pathway, which includes the cAMP, PKA, PKAc and expression of the steroidogenic factor SCARB1 may be impaired. In specific, we measured the expression of SCARB1 in patient RNA extracted by adrenal cortex. The RT-PCR showed a clear downregulation of SCARB1, which is a crucial key factor in steroidogenesis. Moreover, two miRNAs implicated in the epigenetic regulation of SCARB1 have been analyzed in adrenal tissue. Both were found to be downregulated in the patient. This corroborates the hypothesis of a possible compensatory response of the patient’s adrenal cortex to the impaired expression of SCARB1, which would lead to a reduced need of the normal inhibitory effect of the miRNAs on its transcript. This result suggests an alternative possible cause of addisonism in Allgrove syndrome, indicating an impaired expression of nuclear factors in response to the cytoplasmic signal cascade triggered by ACTH. Nevertheless, the specific impairment in glucocorticoid production, rather than a complete adrenal insufficiency, is probably due to the exclusive functional dependency of zone fasciculata to ACTH stimulation [26].

To further dissect this pathway, we evaluated one-point downstream ACTH-cell stimulation such as PKA activation and entrance into the nucleus to enhance *SCARB1* transcription. We found P-PKA protein slightly reduced into the nucleus of patient’s cortical adrenal gland and significantly increased in the cytosolic fraction. This result suggests that PKA or other PKA-shuttling proteins may be possible cargoes needed to be imported into cell nucleus and that this specific mechanism is impaired by damages of the nucleoporin Aladin.

Interestingly, we observed a strongly reduced expression of the ACTH receptor MC2R in patient’s cortical adrenal gland. This result could open different scenarios: either the compensatory response to an ACTH overstimulation because of adrenal gland insufficiency, or the impairment of MC2R expression pathway on cellular membrane. The latter opens an intriguing pathogenic mechanism implying Aladin dysfunction associated to an aberrant nucleocytoplasmic transport of specific transcription factors or transcripts.

The comprehension of the mechanism underlying the adrenal insufficiency in Allgrove syndrome and the elucidation of the possible link with the nucleocytoplasmic transport may represent the initial step for future research and therapeutic approaches in ACTH-resistant hypocortisolism.

## Conclusions

This study provides a unique pathological description of adrenal glands affected by Allgrove's syndrome. The findings could help to shed a light on the mechanism underlying adrenal insufficiency, providing new insight that could link ACTH resistance, SCARB1 impairment, and defective nucleocytoplasmic transport. We aim to confirm the molecular anomalies found in a future biobank in a cohort of several cases of this rare disease. These observations may represent the initial step for future research and new therapeutic strategies for this rare disease.

## Data Availability

Please contact author for data requests.
